# Going to scale—From community-based to population-wide genetic improvement and commercialized sheep meat supply in Ethiopia

**DOI:** 10.3389/fgene.2023.1114381

**Published:** 2023-03-17

**Authors:** Joaquin Mueller, Aynalem Haile, Tesfaye Getachew, Bruno Santos, Mourad Rekik, Berhanu Belay, Dawit Solomon, Likawent Yeheyis, Barbara Rischkowsky

**Affiliations:** ^1^ National Institute for Agricultural Technology (INTA), Bariloche, Argentina; ^2^ International Center for Agricultural Research in the Dry Areas (ICARDA), Addis Ababa, Ethiopia; ^3^ AbacusBio Limited, Dunedin, New Zealand; ^4^ International Center for Agricultural Research in the Dry Areas (ICARDA), Tunis, Tunisia; ^5^ International Livestock Research Institute (ILRI), Addis Ababa, Ethiopia; ^6^ Amhara Regional Research Institute (ARARI), Bahir Dar, Ethiopia

**Keywords:** small ruminants, low-input system, animal breeding, lamb meat, selection, development, benefit

## Abstract

Community-based breeding programs (CBBPs) have shown, at pilot scale, to be effective and beneficial in achieving genetic progress and in improving livelihoods of smallholder communities. In Ethiopia 134 sheep and goat CBBPs were operational producing their own improved rams and bucks. Based on experience the implementation of further programs is possible with appropriate private and public support. A different challenge is the efficient dissemination of the improved genetics produced in current CBBPs to create population-wide economic impact. We present a framework applied to the Ethiopian Washera sheep breed to meet this challenge. We propose the establishment of a genetic improvement structure that supports a meat commercialization model based on the integration of community-based breeding program cooperatives, client communities and complementary services such as fattening enterprises. We calculated that the recently established 28 community-based breeding programs in the Washera breeding tract can provide genetically improved rams to 22% of the four million head. To reach the whole population 152 additional CBBPs are needed. We simulated the genetic improvements obtainable in the current 28 CBBPs assuming realized genetic progress in CBBPs of a similar breed and calculated the expected additional lamb carcass meat production after 10 years of selection to be 7 tons and the accumulated discounted benefit 327 thousand USD. These benefits could be increased if the CBBPs are linked to client communities by providing them with improved rams: additional meat production would be 138 tons with a value of 3,088 thousand USD. The total meat production of the existing Washera CBBPs was calculated at 152 tons and the joint meat production of CBBPs if integrated with client communities would be 3,495 tons. A full integration model, which includes enterprises purchasing lambs for fattening, can produce up to 4,255 tons of meat. We conclude that Washera CBBPs cooperatives can benefit from a higher level of organization to produce population-wide genetic improvement and economic benefits. Unlike in the dairy and chicken industries, for low input sheep and goat smallholder systems the proposed commercialization model puts breeder cooperatives at the center of the operation. Cooperatives need to be capacitated and supported to become fully functional business ventures.

## 1 Introduction

Livelihood of smallholder farmers often relies on their ruminant livestock or poultry. Improving efficiency of smallholder systems is a major aim of research efforts and development projects, which usually focus on issues such as product marketing, pastoral range management, feed production, healthcare and genetic improvement (e.g., Shapiro et al., 2015). The latter includes local breed improvement through pure breeding and introduction of alternative breeds for crossbreeding or breed replacement. In any case improved germplasm, whether in the form of semen, embryos, eggs or live animals, has to be produced and disseminated efficiently. Increasing the productivity of animals through genetic improvement is usually slow and has a small immediate impact but it is cumulative, permanent and can be cost effective. Cost-effectiveness is particularly relevant in low-input systems where cash is needed for immediate household expenses. Genetic improvement programs also encompass non-monetary returns/outcomes. For example, improved livestock may have cultural or social value, new or improved products may contribute to overcome nutritional deficiencies, locally produced additional food may increase food security, more efficient animals may allow a decrease in stocking rates or reduce demand of feed and water, etc. (FAO, 2010).

Implementation of genetic improvement programs in smallholder small ruminant conditions is difficult for several reasons (Wurzinger et al., 2011) and effective programs are very rare. A recent development is the community-based breeding program (CBBP) approach of sheep and goat genetic improvement (Mueller et al., 2015). In these programs instead of focusing on the genetic improvement of the individual household flock or on external sire providers, the focus is on communities where small ruminant keepers agree on cooperating to produce their own improved sires. In Ethiopia 134 CBBPs were operational with different sheep and goat breeds. Each CBBP organized itself as a cooperative, designated enumerators and made them responsible for data collection. Local researchers were trained in data processing. CBBPs proved to work well but required support with seed funding for the revolving expenses, training, recording, breeding value estimation and other knowledge transfer associated costs (Haile et al., 2019). Support from public and private funding organizations allowed replication of pilot CBBPs in several locations and the pilot phase concluded that CBBPs are an effective and beneficial strategy to achieve genetic progress and to improve livelihood at community level (Haile et al., 2020a).

An additional challenge and opportunity is the efficient dissemination of the improved genetics produced in current individual CBBPs to create population-wide impact. Assuming demand for sheep and goat products continues to grow, a new supply chain structure will benefit from the support provided by CBBPs role of genetic improvement providers, i.e., breeding cooperatives, supplying improved sires to client communities which benefit from the higher productivity of their animals and can concentrate on efficient meat production within their smallholder systems. Such a structure would resemble pyramidal genetic structures known to work in developed countries. Development of a more structured and commercially-oriented small ruminant meat supply chain on a large scale requires a conceptual framework and several enabling activities. In this paper we present a framework applied to a specific sheep population, the Ethiopian Washera sheep breed. We proposed the necessary steps to establish a genetic improvement structure that supports a meat commercialization model based on the integration of CBBP cooperatives, client communities and complementary services such as fattening enterprises. The paper also demonstrates the potential impact, both genetic and the expected economic benefit, when a large proportion of the total sheep breed population is influenced by improved local genetics, and discusses implementation issues.

## 2 Materials and methods

### 2.1 Theoretical framework

The experience in established CBBPs demonstrates that selected surplus males become of increasing interest by neighboring farmers and communities to be used for breeding (Abate et al., 2020). Thus, in order to reach a large proportion of a small ruminant population with improved genetics, Mueller et al. (2019) suggested three strategies: 1) substantially increasing the number of male lambs sold for breeding per CBBP (up-scaling), 2) increasing the intensity of use of selected rams by means of artificial insemination (AI) and, 3) further replication of CBBPs (out-scaling). A theoretical analysis concluded that up-scaling the number of improved males from current CBBPs for dissemination and out-scaling current CBBPs are highly feasible strategies for population-wide genetic improvement (Mueller et al., 2019). The more intense use of rams using AI was not cost-effective and was only justified in specific circumstances. For example, sires with exceptional high and accurate breeding values may be used as foundation sires for new CBBPs. Thus, Washera up-scaling and out-scaling strategies were analyzed.

Recent experiences demonstrated the financial feasibility of fattening Horro and Bonga sheep (Zemedu et al., 2018). In the Washera area, the Ethiopia Livestock and Fishery Sector Development Project (LFSDP Regional PCU, 2022) established four cooperatives, each of them with a capacity to fatten 200 lambs at a time and three rounds of fattening per year or 600 lambs per year. There are also about ten other common interest groups with a fattening capacity of 150 lambs each, making a total capacity of 3,900 (4 × 600 + 1,500) lambs in fattening stations per year. The sheep fattening activity is becoming an interesting business opportunity for local development as it requires additional feed production facilities which have been established in the area. These sheep fattening developments were considered as part of an integrated sheep meat production structure.

### 2.2 The Washera sheep breed and current CBBPs

**FIGURE 1:**
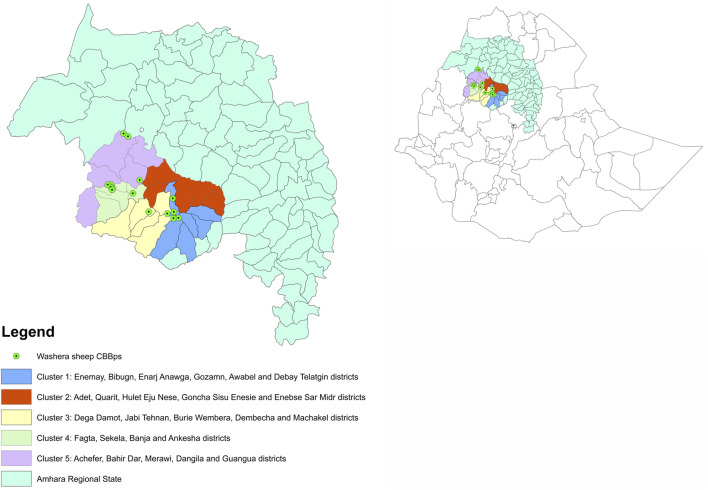
Position of Amhara Regional State in Ethiopia, Washera sheep breed distribution and clusters and location of the current 28 CBBPs.

Washera, also known as Agew or Dangla, is short fat tail; large body size; short-haired; predominantly brown; both males and females are polled; reared by Amhara and Agew communities in Ethiopia (Gizaw et al., 2008). The breed is predominantly distributed in West Gojjam, East Gojjam and Awi zones in the Amhara Regional State in Ethiopia (Figure 1) and is one of the most popular and well-known breeds in the country with a total population of approximately four million heads in about 300 thousand households living in 2,800 communities. The area is well known for having good to very good agricultural potential and the three zones produce substantial surpluses that are sold to other areas and are important for the food supply of the country as a whole. Agriculture, both crop and livestock are the backbone of region’s economy and 85% of the population in the area depending on agriculture (Getahun and Shefine, 2015). Sheep are an important source of income and livelihoods for the local farmers with a potential to support the national economy because of its fast growth potential. Ewe mature weight is in the range of 27–31 kg. The breed is renowned for being prolific and fast growing. These features are highly appreciated in the region and are preferred breeding goals of local farmers. Washera sheep are also regularly used to improve other indigenous sheep breeds, typically in the Amhara region. Despite its importance, before the recent CBBPs there were no formal Washera sheep genetic improvement programs in the country.

The pilot Washera CBBPs were established in 2021 through a typical approach in which the community establishes a cooperative where members formally agree on breeding goal, recording and selection procedures. Farmers with promising male lambs are paid to retain these lambs till final selection as replacement rams using a revolving fund which is cashed once these sires are cast for age and sold for meat. All unselected male lambs are culled for meat. In 2022 there were 28 community-based breeding initiatives covering several districts in five clusters (Figure 1) which were defined by taking into account availability of partners, geographic location, political administration, ram sharing potential, homogeneity in agro-ecology and the location of the implementing institutions.

A recent survey (Washera CBBP Survey, unpublished) indicates a total of 3,233 households engaged in the CBBPs with a mean flock size of 12.08 sheep including 6.73 breeding females. The total number of sires was estimated assuming communities follow the suggested mating ratio of 25 breeding females per sire and a serving period of 2.5 years. A ram survival rate of 0.95 was assumed. The total number of lambs produced were calculated assuming a conception rate of 0.9 and survival to selection or culling age 0.9 and a lambing interval of 8 months, all assumptions were based on field data. The figure for litter size (lambs born per ewe lambing) was taken from survey results in each cluster. Thus, assuming all female lambs and only replacement male lambs are kept for breeding, then a total of 17,154 Washera lambs are annually culled for meat (Table 1).

**TABLE 1:** Current community-based breeding program (CBBP) statistics per geographical production cluster.

Cluster	CBBPs	Total house-holds	House-hold flock size	Ewes per house-hold	Litter size	House-holds per CBBP	Ewes per CBBP	Total ewes in CBBPs	Total sires in CBBPs	Young sires for CBBPs	Lambs culled for meat
	no	no	no	no	no/lambing	no	no	no	no	no/year	no/year
1	7	875	19.71	9.71	1.51	125	1,214	8,500	340	143	7,654
2	9	1,179	9.82	5.55	1.28	131	726	6,538	262	110	4,974
3	2	200	6.00	3.83	1.10	100	383	767	31	13	499
4	5	600	10.00	6.57	1.18	120	789	3,943	158	66	2,760
5	5	379	8.00	5.33	1.06	76	404	2,020	81	34	1,267
Weighted average			12.08	6.73	1.32	115	777				
Total	28	3,233						21,768	871	367	17,154

### 2.3 Meat production and commercialization model

The Washera meat production and commercialization model is proposed by combining existing up-scaling and out-scaling CBBP approaches. In this model, CBBPs produce improved breeding males for client communities, here called production units, which produce the bulk of lambs for meat and lambs for individual fattening enterprises or fattening cooperatives. In the proposed model CBBP cooperatives are the key organizations establishing the necessary business links between smallholder farmers in the village and market players who supply consumers. Thus, establishing a commercialization model of large scale that integrates production units, fattening enterprises, and supply of lambs to the live market or for processing in slaughter houses *via* the CBBP cooperatives.

The integration strategies were modelled with assumptions on numbers of potential ram lambs available for production units, proportions of lambs produced in these units sold for meat or diverted to be finished in individual fattening enterprises or cooperatives as well as number of additional CBBPs required to impact the whole Washera population. The meat production and commercialization model were parameterized such that a range of situations could be tested to predict industry scale, genetic progress and economic impact.

The analyses were done considering a planning period of 10 years and the following three levels or scenarios of integration.

a) Non-integrated scenario–the current situation where all surplus male lambs in CBBPs are culled for meat. No formal integration between CBBPs and production units or fattening enterprises and cooperatives.
b) Partially integrated scenario–above average CBBP male lambs are supplied for breeding in production units. No integration with fattening stations is considered in this case.
c) Integrated scenario–CBBPs supply rams to production units and these supply lambs to fattening enterprises with three fattening capacity options (see details in 2.5).

### 2.4 Calculation of genetic progress

Washera sheep provide meat for sale and consumption. Hence, sustained improvement of litter size (LS) and lamb weight (SMW) are obvious breeding goals. Selected animals should also be adapted to their production systems, particularly resilient to the environments and regular climatic hazards which may be exacerbated by climate change.

Genetic progress for these traits has not yet been calculated from field data in Washera CBBPs. In order to get an estimate of performance in current Washera sheep CBBPs, the genetic progress obtained in CBBPs of Horro breed was used. This breed has similar performance characteristics as Washera, for example, average litter size in Horro is 1.36 lambs/lambing and average lamb weight is 20.0 kg (Zemedu et al., 2018) while for Washera sheep mean ± SD are 1.32 ± 0.34 lambs/lambing and 19.77 ± 3.87 kg, respectively (recent field survey data, n = 437). Also selection procedures applied in both breeds are similar. In Horro, annual genetic progress achieved in SMW was 0.1800 kg/year and progress in LS was 0.0021 lambs/lambing over the period 2009–2018 (Haile et al., 2020a). These improvement rates were applied over 10 years to simulate expected improvement in newly established Washera CBBPs using gene flow methodology (Amer, 1999; FAO, 2010). Genetic progress at production units was calculated considering the average merit of selected males, assuming a parent average approach in which half of their genes and half of average production unit ewe genes are expressed in each year’s progeny batch (Santos et al., 2017).

Potential annual genetic progress in both SMW and LS was calculated using selection index theory (Hazel, 1943) and assuming the Horro sheep parameters, heritability of 0.4090 for SMW and 0.0515 for LS (Kebede, 2002; Haile et al., 2020b) and phenotypic and genetic correlations between SMW and LS of −0.0828 and 0.0340, respectively (Kebede, 2002). Selection differential was obtained considering selection of the top 10% of male candidates and no selection of females (average standardized selection intensity of 0.877) and generation length was estimated to be 3 years. These figures together with the market price of a 20.0 kg Washera lamb of 90 USD and about the same number of expressions for both traits allow calculation of standard index weights and potential genetic progress in both SMW and LS.

### 2.5 Calculation of meat production

Meat production was calculated for each production chain integration level by calculating the number and weight of male lambs culled or male lambs fattened considering dressing percentages of 44.2 and 49.48, respectively (Getachew et al., 2011). For the non-integrated scenario of the present 28 CBBPs, statistics from Table 1 were used. For the partially-integrated scenario, the remaining number of lambs culled for meat in CBBPs and the total number of lambs produced in production units were calculated and multiplied by the average lamb weight of 20.0 kg.

For the integrated scenario which also considers lamb fattening enterprises, three cases (c1, c2, and c3) were calculated: considering the current fattening capacity of 3,900 lambs (c1), increasing fattening capacity at the current rate of 1,000 more lambs a year up to 13,900 at year ten (c2), and increasing fattening capacity up to all acceptable (above average) lambs for fattening (c3). Following the financial feasibility analysis of Zemedu et al. (2018) with fattening of Horro male lambs, an average growth of 8 kg live weight after 90 days fattening period was assumed and survival of lambs in fattening stations of 0.95.

The slight but sustained increase in number of lambs and lamb weight due to genetic improvement of LS and SMW were also considered when calculating meat production but reported separately from a scenario of no genetic improvement. Sensitivity of the assumed rates of genetic progress in LS and SMW on meat production and economic outcome was tested setting rates to 80% and 120% of those observed in Horro CBBPs.

### 2.6 Calculation of economic parameters

Economic benefit of the different scenarios was calculated as revenue minus cost over a 10-year planning horizon assuming a discount rate of 0.07 as in previous studies (Mueller et al., 2019) to make revenues and costs comparable. In the non-integrated scenario the economic benefit from sale of lambs culled for meat was calculated based on lamb numbers and lamb weights and from known genetic trends for LS and SMW. Revenue per 20.0 kg Washera lamb was assumed at 90 USD or 4.5 USD per kg live weight. Several initial and annual costs were considered when establishing a new CBBP. Initial year costs due to the construction of a collection yard, purchase of a scale and ear tag applicator and training or meeting expenses were 700 USD, annual costs due to purchase of ear tags and payment of enumerator were 900 USD as in Mueller et al. (2019). Note that only additional income due to genetic improvement and only additional costs due to the selection program were considered, capital expenses were ignored.

In the partially-integrated scenario the economic benefit at CBBP level was calculated as before but considering that a proportion of lambs are sold for breeding and all male lambs in the production units are culled for meat. The only additional cost considered for production units was an overprice of purchased young CBBP rams. This additional cost for production units and additional income for CBBPs was taken as equivalent to 1 kg live lamb price (4.5 USD). The light annual increase in lamb weights and number of lambs due to genetic improvement were considered and reported separately.

In the three cases of fully integrated systems, those including fattening enterprise, the benefit per kg “finished” meat was calculated as the difference between meat market price minus fattening cost per kg. Costs included veterinary services and other associated costs such as concentrate feeding and watering troughs. This cost per kg was assumed to be 65% of its price, a figure obtained as average cost in two fattening experiments applied to young CBBP Horro rams (Zemedu et al., 2018).

The financial analysis for the three integration scenarios does not include other expenses than those related to the linking and fattening. Selection costs were considered for CBBPs since there would be no CBBP without selection and fattening costs didn’t include basal feed costs. Economic parameters were calculated separately with and without genetic improvement. selection costs, income from sale of cull lambs and income from sale of lambs for breeding were considered for CBBPs; costs due to purchase of breeding lambs, income from sale of lambs either for meat or for fattening were considered for production units; and costs due to purchase of lambs for fattening, costs of fattening and income from sale of fattened lambs were considered for fattening enterprises. Costs and incomes were discounted and accumulated to year 10, when c2 and c3 reach their fattening capacity target. Annual and accumulated discounted revenues, costs and benefits were obtained for the three integration systems at each production tier. Return on investment (ROI) was calculated as accumulated discounted revenues over accumulated discounted costs.

Meat production potential and economic impact were based on the current number of Washera CBBPs. The number of additional CBBPs required to reach the entire Washera population with improved males was extrapolated from the current landscape.

## 3 Results

### 3.1 Genetic improvement of CBBPs and production units

**FIGURE 2:**
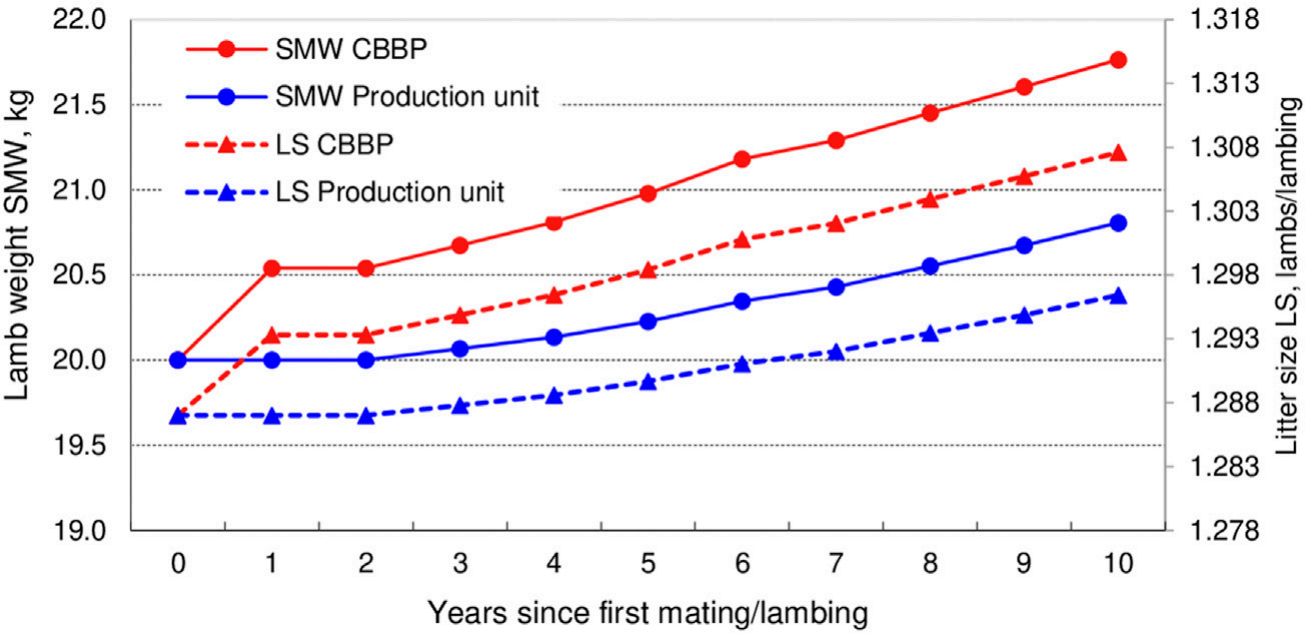
Genetic progress of lamb weights (SMW) and litter size (LS) in CBBPs and client production units starting selecion in year) and assuming no initial genetic differences between CBBPs and production units.

After 10 years of selection in CBBPs the increase in SMW was 1.77 kg and in LS 0.021 lambs. First selected progeny in the CBBPs was born in year 0 (expressing superiority at lamb age, year 1) and first improved males are used in production units in year 1 with improved progeny expressing superiority in year 2. In the early years SMW and LS trends improve rapidly in the CBBPs and the genetic trends in the production units follows with a delay (lag) achieving at year 10 about half the improvement of the ram provided by the CBBPs. The increase in litter size at year 10 implied additional 272 and 1,676 lambs in CBBP and client production units, respectively (Table 4). At year 10 production units lag about two generations of improvement behind CBBPs (6 years), as presented in Figure 2.

### 3.2 Up-scaling washera CBBPs

In the current non-integrated situation, the 28 Washera CBBPs produce young replacement males for own use and all surplus male lambs (17,154) are culled for meat. In the partially integrated situation all CBBP lambs above average (8,322), excluding 5% of lambs culled for physical appearance are considered available for breeding. From these, 367 are used as own CBBP replacements and the remainder 7,956 are selected for use in production units. CBBP lambs not used for breeding (9,198) are culled for meat (Table 2). This amounts to about 2.8 (9,198/3,233 households) lambs culled for meat and about 2.5 (7,956/3,233) young sires sold for breeding per CBBP household per year. In addition, there is a potential for another 193,063 lambs to be made available for fattening.

**TABLE 2:** The effect of CBBPs sire production capacity on production units, meat lamb and potential fattening lamb numbers. Estimated CBBP out-scaling requirements.

Cluster	Sire prod. Capacity in CBBPs	Young sires for prod. Unit	Lambs culled for meat in CBBP	Ewes in prod. Unit	Meat lambs in prod. Unit	Potential lambs for fattening	Potential finished lambs	Targeted ewes in cluster	Gap of ewes	Gap of CBBPs
	no/year	no/year	no/year	no	no/year	no	no/year	no	no	no
1	3,704	3,561	4,094	211,407	193,929	96,965	92,116	147,844	−63,563	−2
2	2,415	2,305	2,669	136,847	106,412	53,206	50,546	1,092,459	955,612	60
3	243	230	269	13,682	9,143	4,572	4,343	155,874	142,192	20
4	1,343	1,276	1,484	75,772	54,317	27,158	25,800	260,948	185,176	12
5	618	584	683	34,667	22,324	11,162	10,604	490,750	456,083	62
Total	8,322	7,956	9,198	472,376	386,126	193,063	183,410	2,147,875	1,675,499	152

According to data compiled from the regional livestock office the total Washera ewe population in the five production clusters was 2,147,875. With current up-scaled CBBPs only 22% (472,376/2,147,875) of the ewes in this population could be served with CBBP born rams. Assuming that new CBBPs in each cluster will be of the average CBBP size in the cluster, it was estimated that 152 additional CBBPs are required to serve the whole Washera ewe population. In cluster one there are no more CBBPs needed, in fact with the proposed up-scaling strategy there are rams in excess to serve 63,563 additional ewes at another CBBP site or in production units. On the other hand, in cluster 5, 62 additional CBBPs are required to cover the target population of 490,750 ewes (Table 2). Clearly, these figures are indicative and open to arrangements between cooperatives of the different clusters. In any case the results show the need for specific extension services and CBBP promotion in each district.

### 3.3 Meat production

Current annual meat production of the 28 CBBPs is about 152 tons (carcass weight) and in the partially integrated scenario 3,495 tons, 81 tons from culled lambs in CBBPs and 3,413 tons from culled lambs from production units (Table 3). Note here and elsewhere in tables and text minor rounding effects in the report of numbers. The effect of genetic improvement in number of lambs and in lamb weights results in an increase of 7.4 tons in CBBPs and 138 tons in production units at year 10 (Table 4). This amount of additional meat due to genetic improvement is expected if genetic progress in the recently established Washera CBBPs achieves the progress obtained over 9 years in Horro CBBPs, that is 0.18 kg/year in SMW and 0.0021 lambs/lambing in LS. Using an index based on Horro and Washera parameters (section 2.4) would increase SMW progress to 0.46 kg/year and would increase meat production accordingly. In the fully integrated scenario, another 51 tons of finished lamb meat is produced using the current lamb fattening capacity (c1). Assuming a growing fattening capacity, fewer lambs are culled for meat but more are fattened. Considering the final (year 10) target capacity of 13,900 lambs (c2) and the maximum number of lambs available for fattening, 193,000 lambs (c3), a range between 183 tons and up to 2,541 tons finished carcass meat can be produced. The sensitivity test shows that the additional meat production is directly proportional to the assumed rates of genetic progress. If these rates in Washera CBBPs would be only 80% of those achieved in Horro CBBPs the total additional meat production would be 116 tons instead of 146 tons and if these rates would be 120% then the total additional meat production would be 175 tons (Table 4).

**TABLE 3:** Yearly lamb and carcass meat production in three integration scenarios of CBBPs, production units and fattening enterprises, excluding the effect of genetic improvement. Based on current 28 CBBPs and three levels of expected final fattening capacity (c1, c2, and c3) described in section 2.5.

Parameter	Unit	Non-integrated (CBBP)	Partially integrated (CBBP + production unit)			Integrated (CBBP + production unit + fattening enterprise)		
						c1	c2	c3
		CBBP	CBBP	Production unit	Total	Total	Total	Total
Number of lambs culled for meat	no	17,154	9,198	386,126	395,324	391,424	381,424	202,261
Number of finished lambs	no	0	0	0	0	3,705	13,205	183,410
Lamb carcass meat	tons	152	81	3,413	3,495	3,415	3,306	1,714
Finished carcass meat	tons	0	0	0	0	51	183	2,541
Total carcass meat	tons	152	81	3,413	3,495	3,466	3,489	4,255

**TABLE 4:** Predicted genetic merit in CBBP and client production units after 10 years of selection for simultaneous improvement of lamb weight and litter size and its effect on additional meat production and economic benefit. Assumed rates of genetic improvement in lamb weight and litter size are those observed in Horro sheep CBBPs.

Parameter	Unit	Assumed rates of genetic improvement				80% of assumed rates of genetic improvement				120% of assumed rates of genetic improvement		
		CBBP	Prod. Unit	Total		CBBP	Prod. Unit	Total		CBBP	Prod. Unit	Total
Initial lamb weight	kg	20.0	20.0			20.0	20.0			20.0	20.0	
Initial litter size	lambs/lambing	1.287	1.287			1.287	1.287			1.287	1.287	
Final lamb weight	kg	21.77	20.81			21.41	20.65			22.12	20.97	
Final litter size	lambs/lambing	1.308	1.296			1.303	1.295			1.312	1.298	
Additional lambs	no	272	1,676	1,949		218	1,341	1,559		327	2,012	2,338
Additional carcass meat	tons	7.4	138	146		5.9	111	116		8.9	166	175
Accumulated discounted income	000′$	549	3,340	3,889		489	2,670	3,159		610	4,009	4,619
Accumulated discounted cost	000′$	222	251	473		222	251	473		222	251	473
Accumulated discounted benefit	000′$	327	3,088	3,415		267	2,419	2,686		388	3,758	4,146
Return on investment	$/$	2.5	13.3	8.2		2.2	10.6	6.7		2.7	15.9	9.8

### 3.4 Economic benefit for CBBPs and production units

**FIGURE 3:**
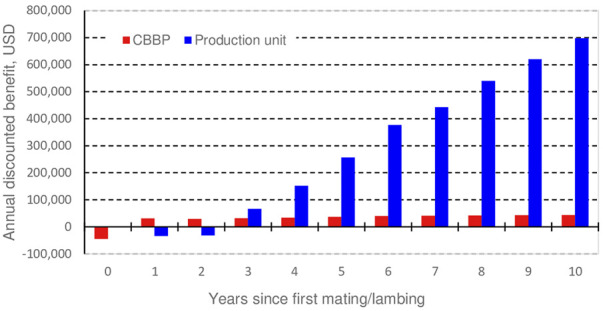
Additional annual discounted benefit in current CBBPs and production units. Selection starts at year 0. For CBBPs benefit becomes positive in year one mainly due to sale of breeding males to production units. For these, benefit becomes positive in year three and increases as improved genes flow through the population.

The additional annual discounted benefit due to genetic improvement of SMW and LS in 28 CBBPs and their client production units over 10 years of selection is shown in Figure 3. The selection program in the CBBPs with first progeny born from selected parents starts in year 0 and the purchase of rams by production units starts in year 1. In year 0 CBBPs face initial costs with no economic benefit, but through the following 10 years CBBPs profit from higher lamb weights and sales of young rams to production units. Production units start to have expenses in year one when buying first improved young rams. At year three these expenses are compensated with increased lamb numbers and increased lamb weights. Due to the large number of ewes in the production units (472,376 ewes) the total benefit is much higher than in the CBBP level.

Return on investment (ROI) for the improvement program in the CBBP layer is much lower than in the production unit layer (2.5 vs. 13.3 USD per USD invested, Table 4) creating opportunity for a higher CBBP ram lamb price offered to production units. This might be contemplated once the improvement programs advance, and these ram lambs clearly stand out from those currently used. The ROI would also be much higher with more years of improvement and if we assume initial genetic differences in SMW and LS between CBBPs and production units. This is relevant in the future as CBBPs progress and new production units joining the integrated chain. Note also that the accumulated discounted benefit is directly proportional to the rates of genetic improvement assumed (Table 4).

Benefit for CBBPs integrated with production units resulted slightly greater than benefit for CBBPs in the current in the non-integrated situation. The combined benefit for CBBPs and production units is more than 20 times higher although this isn’t really an additional benefit to the benefit a similar number of unlinked farmers would have. The non-accounted benefit will be more related with genetic improvement and marketing capacity when joining the integration chain. Depending on the fully integrated scenario (c1, c2 or c3) more meat is produced (Table 3) but only a slightly higher benefit is obtained due to high costs to produce fattened meat. However, combined ROI are high for all scenarios (Table 5).

**TABLE 5:** Financial analysis of three integration scenarios between CBBPs, production units and fattening enterprises. Integration scenarios c1, c2, and c3 involve increasing lamb fattening capacities (see text for a detailed description). Total refer to the sum of CBBP, production unit and fattening enterprise.

		Non-integrated	Partially integrated			Integrated		
						c1	c2	c3
Economic parameter	Unit	CBBP	CBBP	Production unit	Total	Total	Total	Total
Accumulated discounted income	000′$	12,387	12,675	278,830	291,505	295,250	299,409	372,251
Accumulated discounted cost	000′$	222	222	5,280	5,502	9,051	12,990	81,999
Accumulated discounted benefit	000′$	12,166	12,453	273,549	286,002	286,199	286,418	290,252
Return on investment	$/$	55.9	57.1	52.8	53.0	32.6	23.0	4.5

## 4 Discussion

### 4.1 Model and alternatives

We proposed a framework to impact the Washera sheep population with genetic improvement in litter size and lamb growth rate, affecting its entire lamb meat supply chain. The underlying structure is a pyramidal genetic structure where CBBPs cooperatives are the nucleus responsible for sustained genetic improvement and delivery of improved rams to production units. Eventually, genetic progress achieved in CBBPs reaches a large proportion of the total Washera population through reduction in genetic lags between tiers and through sustained improvements supported by current and future CBBPs. The proposed genetic improvement and dissemination model leading to a novel meat supply chain differs from models applied in the poultry and dairy cattle industry.

In Ethiopia, experiences with improved layer and broiler chicken breeds, in particular with dual purpose breeds or crossbreds between commercial and indigenous breeds, as well as their dissemination, has been in place for a long time. The dynamics of genetic improvement is “facilitated” since production of genetically improved eggs and chicks can be centralized and performed on a large scale. Improved eggs and chicks can be easily transported and distributed, but mostly under better control of specialized suppliers. The productive impact of improved poultry germplasm is almost immediate because broiler growth or laying hens production is quickly realized and generation length is short. Projects facilitating farmer’s access to preferred locally adapted improved breeds and a sustained multiplication and delivery system together with feed and health services are already in place (Sartas et al., 2021). These schemes are managed by private companies and are common in many developing countries.

Dissemination of improved dairy cattle genetics has also been experienced for a long time in Ethiopia and in many African countries. In this case, generation length is high, but improvement can be channeled through artificial insemination (AI) centers where proven bulls (often imported from developed countries) provide semen to be used on site to inseminate local cows. Performance testing and pedigree recording is well established in many dairy industries, so that information can be processed using BLUP breeding values in order to detect young dairy bull candidates for the AI center. Thus, a business structure has evolved around cattle AI centers providing semen doses and insemination services (Marshall et al., 2019).

In most developing countries, breeding and genetic improvement services in sheep and goat are rather uncommon and are more complicated to apply compared to poultry or dairy cattle. In many developed countries population wide genetic improvement of sheep and goats relies on effective pyramidal genetic structures with a stud (nucleus) tier producing males for multipliers and these producing males for the base (commercial) population. Fresh and frozen semen AI services are available and progeny testing facilities as well as sire referencing schemes are offered. Controlled matings and systematic performance recording structures allows both within and across breed genetic evaluations and optimum use of genetic variability.

In low-input smallholder situations such as in Ethiopia, pyramidal systems and structured crossbreeding are difficult to implement. Communal grazing of pastoral systems and limited infrastructure to control matings is challenging and therefore full pedigree recording is often impossible. This limits the ability to run proper population-wide BLUP evaluations. Insemination with frozen semen in sheep is also more challenging compared to other species and much costlier than in cattle since it requires laparoscopic AI instead of intrauterine non-surgery procedures. Moreover, farmers in smallholder systems have no easy access to improved genetics. Dedicated producers of locally adapted breeds are scarce. Frequently when improved genetics is available, it is from exotic breeds which in most cases are not at all adapted to smallholder system or environmental conditions faced locally.

A strategy to overcome at least partially these limitations is to concentrate breeding activities in public research stations and distribute improved males to private farmers or communities (Kosgey et al., 2006). There are concerns on the suitability of the particular breeding objectives, proper management and the actual genetic progress achieved in such governmental stations and the adaptation of station bred sires to perform in smallholder farmer environments. The main problem with this strategy is its dependence on the particular institutional funding policy and the risk to lose centralized structures due to natural disasters, disease outbreaks and conflict situations. For example, the Abergelle goat nucleus of the Sekota Dryland Research Center was lost due to the conflict in Northern Ethiopia. The consequence of these limitations with traditional pyramidal structures and centralized nucleus is that in countries like Ethiopia formal within breed selection programs for sheep and goats are rarely found.

The CBBP experience and its organization as cooperatives is conductive to solving most business-related issues and encourage genetic improvement limitations (e.g., pedigree and genetic evaluation structures) to be addressed. A pyramidal structure can be emulated through the proposed out-and up-scaling strategy and integration of CBBPs, production units and fattening enterprises. The proposed framework overcomes key issues related to stakeholder roles, breeding goals, meat production scaling strategy, sustainability, resilience and independence. There are, of course implementation issues and areas for further adjustments and research needs.

Finally, one of the biggest limitations that is minimized with the CBBP framework is that of ownership and funding of the breeding scheme. In this approach, ram lambs selected and commercialized under the breeding cooperative structure proposed, create enough revenue to support its maintenance and allow running costs to be met. There is also a significant opportunity to leverage this investment, made by the farmers themselves *via* improved rams and cooperative arrangements, through fattening and supplying finished lambs to better paying markets. Other collective arrangements may include lamb conditioning initiatives to provide export slaughter houses with appropriate lambs. Ultimately CBBPs can be seen as a “starting-point for initiators and participants to continuously discover new ways of collaboration and engagement” (Wurzinger et al., 2021).

### 4.2 Implementation of the model

#### 4.2.1 How to make it work

Predictions of meat production and economic benefits were based on current 28 Washera CBBPs reaching an estimated 22% of the total Washera sheep population. To reach the whole Washera population additional 152 CBBPs would be needed. Experience for establishing new CBBPs has accumulated and guidelines for this task are available (Haile et al., 2018; Mueller et al., 2021). Nevertheless, each new CBBPs face challenges which need to be addressed (Haile et al., 2020b; Endris et al., 2022). The rapid multiplication of pilot CBBPs was largely possible with the joint complementary effort of a number of organizations. Further implementing institutions have to be detected and involved in the establishment of new CBBPs. Additional seed funding will be necessary and a comprehensive training program at community, extension and research centers must be organized and executed. Many more communities need the motivation, incentive and support to agree and organize themselves as a functional CBBP. This requires region-wide awareness and understanding of the CBBP concept. It needs training and technical advice, economic benefit and access to markets.

A key integration factor within the supply chain is the production and dissemination of improved ram lambs for breeding. In some cases, innovative arrangements between CBBP cooperatives and client production units may be found. Outstanding production unit females may be exchanged for selected CBBP males. This would open the nucleus to base population genes. Clients also need access to lamb markets and to fattening. Such complementary business options near the CBBPs were already initiated by various organizations, sometimes involving youth groups. Integrating these groups through genetic dissemination may also be an additional incentive for the establishment of these types of structures across multiple regions. Economic feasibility analyses have shown that fattened males accrued higher net profit than control males in Bonga and Horro sites but were unrewarding in Menz and Doyogena sites (Zemedu et al., 2018). Clearly, fattening depend heavily on supplement costs and economic benefit will depend on the ability to access cheap quality feed and the ability to market better lambs for higher prices. Since feed costs are volatile, close monitoring of economic parameters will be needed.

#### 4.2.2 Increasing rate of genetic progress

The simulations described in this study were based on the recently established Washera CBBPs. For that reason, no difference in initial genetic merit between CBBPs and production units was assumed in this simulation. This might be different in future years when current CBBPs will have improved. In that case genetic progress in production units will be faster, but the initial lag is likely to be larger. To reach the entire Washera population with improved rams, another 152 CBBPs would need to be established and integrated with production units (Table 2). An alternative to guarantee supply of the required number of rams would be by reducing selection intensity of males, that is selecting more than 50% of the available males. But this affects genetic progress and makes selected male lambs less attractive for breeding. Another aspect to look at more carefully is selection efficiency given the much higher progress expected in SMW with efficient index selection. An analyses of the reasons for this difference may give hints to further adjustments of selection procedures. A large scale AI program would also increase the improved population but would require an important public or private financial support, i.e., business opportunities to be undertaken by private-sector. The most prominent solution is to out-scale CBBPs, a strategy which has proven to be sustainable with high ROI but which needs training activities, community engagements, and a highly qualified multidisciplinary team to combine research activities, extension and services to smallholder farmers. In any case, all means to make population-wide impact should be exploited, probably leading to a smart combination of up-scaling, out-scaling and AI opportunities.

Continuous genetic improvement has been achieved following an efficient performance recording protocol integrated with CBBP specific breeding value estimation systems. Breeding values were estimated for each CBBP separately since genetic links between CBBPs are weak or absent. In the long term, farmers would benefit from population-wide genetic evaluations and access to superior males across CBBPs. Such an evaluation is in principle not difficult but needs genetic links which can be created using reference sires, first within clusters and then across clusters. Artificial insemination will be a convenient tool to facilitate this linkage. A centralized database with a unique identification system and recording protocol applied across CBBPs has been progressively implemented (https://dtreo.io/), allowing a population-wide genetic evaluation to be targeted and thereby increasing the access to genetic diversity and consequent genetic progress.

Centralized genetic evaluations require agreements across communities on technical aspects such as measurements, economic weights, use of link sires, etc. It also requires agreements on promotion and marketing aspects. All these call for a close communication between communities which nowadays is facilitated by the increased accessibility to mobile phones and other communication means. Such linking of communities with similar breeding interest also lead to an across CBBP genetic evaluation. Other important cooperation items may include research needs, breed promotion programs, ram sale calendar, AI program, other.

#### 4.2.3 Resilience and sustainability

A concern among livestock breeders is whether their animals are resilient to climate changes. A major advantage of CBBP livestock is that selection of local breeding stock takes place in the same environment where target production takes place. Thus, adaptation genes are secured as local breeds are more resilient (Tibbo et al., 2008). Climate changes are expected to produce more extreme situations and a slow but constant average temperature increase (IPCC, 2022). The integration enabled by the CBBP approach allows the breeding programs to accommodate to changes faced by smallholder farmers as climatic trends cause forage availability limitations, instance. Genetic improvement in this model is a low-cost way for these communities, as improved livestock tend to be more efficient, and the structure of the integrated supply chain allows more or less animals to be diverted to the lamb market or the breeding market accordingly.

In the future, traits susceptibility to climate changes (Berghof et al., 2019) will be included in breeding goals and selection indexes. In particular, health related traits representing the breeding goal and implemented with support from breeding values estimated within the CBBP structure. The financial sustainability of the proposed intervention is also largely guaranteed. Altogether, there are 134 CBBPs operating in Ethiopia, this success rate is partly the result of constructive involvement of all stakeholders and partly because the CBBP establishment isn’t based on large investments nor highly cash dependent. Experiences and lessons collected in Malawi and Uganda also highlight the importance for different actors to work together by pooling financial resources and technical expertise for establishment and sustainability of goat CBBPs (Kaumbata et al., 2020). CBBPs work with locally adapted animals, and therefore, the issue of environmental sustainability is embodied in the CBBP concept (Haile et al., 2019).

The general concept of CBBPs and the proposed framework should be conveyed by credible personnel and institutions, jointly or in close agreement with the national agricultural system (NARS). Involving personnel from a number of institutions, essentially from local agricultural stations and public extension service, is critical for success of such initiatives. This includes a strong collaboration as CBBPs must have a responsible extension officer network. These officers may attend one or more CBBPs and require periodical training related to the implementation, execution and monitoring of CBBPs. Senior extension officers and researchers are in charge of the most technically demanding genetic specific activities such as genetic evaluation, artificial insemination and ram selection. Evaluation of the programs and formal steps to implement adjustments and compliance requirements are also required and available (Lamuno et al., 2018).

#### 4.2.4 Additional innovations and services

The necessary feed resource development, health intervention and market linkages require support and guidance. Such needs should be supported on top of genetic improvement. Under the Ethiopia Small ruminant value chain transformation (SmaRT Ethiopia) program, ICARDA and partners developed a number of innovations, including genetic improvement, dissemination of improved genetics, development of feed and forages, fattening of lambs/kids, animal health interventions, and innovative market outlet with capacity building and innovative credit accessibility through cooperative organization. These innovations were tested in different areas and positive socio-economic benefit reported (Kassie et al., 2021). Pro poor livestock development is about all the components of improvement working in concert and at scale. Therefore, in the proposed genetic improvement scheme, it is imperative that all value chain components are adequately addressed to bring about transformational change.

New research needs arise with implementation. If fattening initiatives multiply genetic improvement goals, CBBPs may need to consider additional traits such as growth rate to finishing weight and feed conversion or residual feed intake, since feeding cost would become an issue.

## 5 Conclusion

CBBPs produced a big impact on livelihood of individual communities. We have shown how individual CBBPs can benefit from a higher level of organization with cooperatives as main actors to achieve genetic improvement at population-wide level and estimated the resulting economic benefits. Through effective integration with fattening enterprises and output markets, this could also lead to more organized structures in the Washera meat supply chains. Institutional efforts focusing on supporting the role of the different tiers of the structure is essential.

## Data Availability

The data analyzed in this study is subject to the following licenses/restrictions: These are breeding data available based on request and permission. Requests to access these datasets should be directed to a.haile@cgiar.org.
